# Efficacy of Silexan in patients with anxiety disorders: a meta-analysis of randomized, placebo-controlled trials

**DOI:** 10.1007/s00406-022-01547-w

**Published:** 2023-01-30

**Authors:** Markus Dold, Lucie Bartova, Hans-Peter Volz, Erich Seifritz, Hans-Jürgen Möller, Sandra Schläfke, Siegfried Kasper

**Affiliations:** 1 Department of Psychiatry and Psychotherapy, Medical University of Vienna, Währinger Gürtel 18–20, 1090 Vienna, Austria; 2 Hospital for Psychiatry, Psychotherapy and Psychosomatic Medicine Schloss Werneck, Balthasar-Neumann-Platz 1, 97440 Werneck, Germany; 3 Department of Psychiatry, Psychotherapy and Psychosomatics, Psychiatric Hospital of the University of Zurich, Zurich, Switzerland; 4 Department of Psychiatry and Psychotherapy, Ludwig-Maximilians-University Munich, Nußbaumstraße 7, 80336 Munich, Germany; 5 Dr. Willmar Schwabe GmbH & Co. KG, Willmar-Schwabe-Straße 4, 76227 Karlsruhe, Germany; 6 Center of Brain Research, Medical University of Vienna, Spitalgasse 4, 1090 Vienna, Austria

**Keywords:** Silexan, Lavender, Anxiety disorders, Meta-analysis, Efficacy, Tolerability

## Abstract

**Introduction:**

We report on a meta-analysis of Silexan, a proprietary active substance produced from *Lavandula angustifolia*, in subthreshold anxiety, mixed anxiety and depressive disorder (MADD), and generalized anxiety disorder (GAD).

**Methods:**

The present analyses are based on all currently completed 5 double-blind, randomized, placebo-controlled trials investigating Silexan in adult out-patients who received Silexan 1 × 80 mg/day or placebo for ten weeks according to random assignment (*n* = 1213). Efficacy was assessed based on the Hamilton Anxiety Rating Scale (HAMA), several anxiety self-rating scales, the Clinical Global Impression (CGI) scale, and the Short Form-36 (SF-36) health status questionnaire.

**Results:**

After ten weeks’ treatment, Silexan was significantly superior to placebo in reducing the HAMA total score (including the psychic and somatic anxiety sub-scores) and self-rated anxiety. Based on a ≥ 50% HAMA total score reduction, the responder rate ratio was 1.34 favoring Silexan, and the rate ratio of subjects much or very much improved according to the CGI was 1.51. Silexan was also significantly superior in improving the physical and mental health summary scores of the SF-36. There were no significant between-group differences concerning the occurrence of adverse events (AEs), serious AEs, and premature withdrawal due to AEs.

**Conclusions:**

This meta-analysis demonstrates that Silexan exerts significant anxiolytic effects in subthreshold anxiety, GAD and MADD that were consistently reflected in investigator ratings and patient-reported outcomes, including improvement of health-related life-quality, while showing favorable tolerability and safety.

## Introduction

Anxiety disorders are the most prevalent psychiatric disorders, the 12-month prevalence of anxiety disorders was estimated at 10.3% and a lifetime prevalence around 34% [4, 7]. While global health, measured by disability-adjusted life-years (DALYs) attributable to a pool of 369 diseases and injuries and standardized for population growth and ageing, has steadily improved over the last 30 years, the standardized rate of DALYs caused by anxiety disorders has remained stable [7]. Thus, their relative importance as a cause of disability has increased. In 2019, diagnosed anxiety disorders were ranked to be the 15th most important non-communicable cause of DALYs in patients of all sexes and ages, the 10th most important in patients between 25 and 49 years of age, and the 3rd most important in patients aged 10–24 years, with higher prevalence rates in females [21]. However, according to an Australian study, only 27% of 966 participants meeting the diagnostic criteria of a past-year anxiety disorder according to the International Classification of Diseases, Tenth Revision (ICD-10) sought the assistance of a health care professional. While 61% of those received an evidence-based intervention, 31% received minimally adequate treatment [27]. These findings are consistent with European data indicating that less than 20% of patients with an anxiety disorder are treated adequately [31]. Since anxiety disorders are associated with a high burden of illness [5, 7], they may have negative consequences, including disability, low life-quality, reduced ability to work leading to loss of productivity, and a high suicidal risk when remaining un- and undertreated [31].

With regard to the pharmacotherapy of anxiety disorders, current international practice guidelines [e.g., 1, 3, 6, 7, 20, 40, 48, 59] mainly recommend selective serotonin reuptake inhibitors (SSRIs–e.g., citalopram, escitalopram, paroxetine, sertraline) and serotonin-norepinephrine reuptake inhibitors (SNRIs–e.g., venlafaxine, duloxetine) as first-line and tricyclic antidepressants (TCAs) and moclobemide as second-line treatments. Further second-line recommendations exist for add-on benzodiazepines in short-term given their potential to develop abuse and dependence [e.g., 5, 9, 55] as well as undesired sedation [5, 34]. The latter adverse effect (AE) refers also to buspirone, representing further second-line psychopharmacotherapeutic option, and partly to pregabalin that can be very effectively applied as add-on treatment in patients with generalized anxiety disorder (GAD).

Due to the potential AEs mentioned above and those of SSRIs and SNRIs, which may include jitteriness, nausea, restlessness, headache, fatigue, altered appetite, weight gain or loss, tremor, sweating, QTC prolongation, sexual dysfunction, diarrhea, and constipation [5], and potential paradoxical and discontinuation symptoms [17, 18, 49], which are, importantly, less pronounced under SSRIs and SNRIs as compared to TCAs [7], the currently recommended medication for anxiety disorders may be suboptimal in terms of tolerability [52}. In turn, the potentially associated reluctance to adhere to the psychopharmacotherapy may explain why anxiety disorders remain un- or undertreated [12]. After years of relative stagnation in anxiolytic drug development [24], a growing interest in the investigation of novel psychopharmaceutic targets has led to an increased investigation and introduction of promising compounds including the effective and well tolerable phyto-pharmacotherapeutic alternative Silexan.

The pharmacological profile of the herbal compound Silexan,^1^ an essential oil for oral administration manufactured from *Lavandula angustifolia* flowers complying with and exceeding the quality definition the monograph Lavender oil of the Ph. Eur. [38, 46], has been assumed to be based on a potent inhibition of voltage-dependent calcium channels (VDCCs) as demonstrated in studies with murine synaptosomes, primary hippocampal neurons and stably overexpressing cell lines [56]. Inhibition of VDCCs is thought to attenuate the overreaching, situationally inadequate stress response of the central nervous system associated with anxiety and mood disorders [53]. Furthermore, Silexan has been shown to significantly increase the density of 5-HT_1A_ receptors and to reduce the serotonin-1A receptor binding potential, leading to increases in extracellular serotonin, dopamine, and norepinephrine [2, 41].

The rapidly growing number of international studies, reviews and meta-analyses indicates a huge interest in lavender oil preparations for the treatment of the so-called subthreshold anxiety [60], anxiety disorders, their frequent comorbidities and related symptoms [8, 58, 61]. In terms of subthreshold anxiety and anxiety disorders, Donelli et al. [15] as well as Sayed et al. [54] reviewed randomized and non-randomized studies investigating different lavender oil preparations with various routes of administration (e.g., oral, aromatherapy, massage oil), while Generoso et al. [22] and Yap et al. [63] focused on Silexan. Hereby, meta-analyses of different strengths of Silexan, that was investigated in one reference-controlled and four placebo-controlled clinical trials in anxiety disorders, were presented. Furthermore, Möller et al. [44] reviewed the effects of Silexan in placebo-controlled trials in subthreshold anxiety disorders. Our research adds to the existing evidence by presenting a meta-analysis of all placebo-controlled, therapeutic trials with the currently marketed dosage of 1 × 80 mg/day Silexan in subthreshold anxiety, mixed anxiety and depressive disorder (MADD) and GAD completed to date.

## Materials and methods

### Study and participant characteristics

A total of five randomized, double-blind, placebo-controlled clinical trials investigating the efficacy and tolerability of Silexan in subthreshold anxiety and anxiety disorders were completed by the manufacturer of Silexan at the end of the year 2020 [33, 35–37, 39]. Additionally, free-text searches of all fields of PubMed as well as of the EU Clinical Trials Register, the ISRCTN Register and of the ClinicalTrials.gov register were performed to identify any other studies with Silexan in subthreshold anxiety and anxiety disorders performed independently of the manufacturer. Search terms were ‘anxiety’ in combination with either ‘Silexan’, ‘Lasea’, ‘WS1265’ or ‘WS 1265’ (‘WS 1265’ was the internal code used by the manufacturer for Silexan) and suppressing the automatic PubMed translation of ‘Silexan’ to ‘lavender oil’ when building the search query. Searches were performed from the earliest record until December 2022. Searching PubMed resulted in 37 matches, none of which referred to a double-blind, randomized, placebo-controlled, therapeutic clinical trial with Silexan in subthreshold anxiety and anxiety disorders beyond those already mentioned. Searches in the indicated trial registers also did not add any clinical trials meeting these criteria.

The 5 trials included into our analysis were performed according to essentially similar protocols that differed mainly in the diagnosis for inclusion and in the derived inclusion and exclusion criteria as well as in some secondary outcome measures (Table 1). Trial A reported by Kasper et al. [35] (trial A) assessed patients with subthreshold anxiety, Kasper et al. [33] (trial B) investigated patients with restlessness and sleep disturbances, the trial of Kasper et al. [39] (trial C) was performed in patients with MADD, and Kasper et al. [37] (trial D) as well as Kasper et al. [36] (trial E) investigated patients suffering from GAD. In all trials, the participants were male or female out-patients between 18 and 65 years of age who sought treatment by a psychiatrist or by a general practitioner. In addition to meeting the diagnostic criteria for inclusion shown in Table 1, eligible participants had to have a baseline Hamilton Anxiety Rating Scale (HAMA; [26]) total score ≥ 18 points and had to meet other anxiety specific eligibility criteria as shown in Table 1.

**Table 1:** Main study design characteristics and subject inclusion criteria

Trial	A (Kasper et al. [35])	B (Kasper et al. [33])	C (Kasper et al. [39])	D (Kasper et al. [37])	E (Kasper et al. [36])
Design characteristics	Double-blind, randomized, placebo-controlled, multicenter, parallel-group				
Diagnosis for inclusion	Anxiety disorder not otherwise specified (DSM-IV 300.00; ICD-10 F41.9)	Restlessness and sleep disturbances (ICD-10 R45.1)	Mixed anxiety and depressive disorder (ICD-10 F41.2)	Generalized anxiety disorder (DSM IV 300.02, also corresponding to DSM-5 criteria; ICD 10 F41.1)	
Anxiety specific selection criteria	HAMA total score ≥ 18 points; HAMA items ‘Anxious mood’ and ‘Insomnia’ ≥ 2 points	HAMA total score ≥ 18 points; HAMA items ‘Tension’ and ‘Insomnia’ ≥ 2 points	HAMA total score ≥ 18 points; HAMA item ‘Anxious mood’ ≥ 2 points	HAMA total score ≥ 18 points; HAMA items ‘Anxious mood’ and ‘Tension’ ≥ 2 points; CAS total score ≥ 9 points	HAMA total score ≥ 18 points; HAMA items ‘Anxious mood’ and ‘Tension’ ≥ 2 points; HAMA sub-score ‘Psychic anxiety’ ≤ 21 points; CAS total score ≥ 9 points
Interventions	1 × 80 mg/day Silexan or placebo^a^, 10 weeks				
Primary efficacy outcome measure	HAMA total score change between baseline and end of treatment				
Other scales assessed for efficacy	SAS, CGI, SF-36	SAS, CGI	HADS, CGI, SF-36	CAS, CGI, SF-36	CAS, CGI, SF-36

*HAMA* Hamilton Anxiety Rating Scale [26], *SAS* Zung Self-Rating Anxiety Scale [66], *HADS* Hospital Anxiety and Depression Scale [65], *CAS* Covi Anxiety Scale [13], *CGI* Clinical Global Impressions [48], *SF-36* Short Form (36) Health Survey [62]

^a^In addition to Silexan 80 mg/day, trial D included treatment groups receiving Silexan 10 or 40 mg/day, and trial E included groups that received Silexan 160 mg/day or paroxetine. Results for Silexan other than those for the marketed dosage of 80 mg/day or for active comparators are not covered in this work

The schedule of each trial started with a 3–7-day qualification phase after which eligible subjects entered a 10-week double-blind, randomized treatment phase. Eligibility criteria had to be met both at the start (screening) and at the end of the qualification phase (baseline). In trials A, B, D, and E, post-baseline outcome assessments were scheduled every two weeks while the protocol of trial C included assessments at the end of weeks one, two, four, seven, and ten.

### Interventions

Participants took Silexan or matching placebo for 10 weeks. Treatments were available in immediate-release soft gelatin capsules. Silexan is a proprietary essential oil manufactured from *Lavandula angustifolia* flowers by steam distillation that complies with the monograph Lavender oil of the European Pharmacopoeia and exceeds the quality requirements of the monograph. Batch to batch consistency is assured by a well-defined, standardized manufacturing process. To avoid inadvertent unblinding, the smell of the capsules containing Silexan was matched by flavoring the capsules containing placebo with 1/1000 of the amount of lavender oil contained in the Silexan capsules.

Analyses were performed on trial participants who received either the recommended daily dose of the marketed product, i.e., 1 × 80 mg Silexan, or placebo. Trial D also included additional treatment arms with 10 and 40 mg/day Silexan. In trial E, paroxetine served as an active control, and another group received Silexan 160 mg/day. The results of these treatment groups were not considered in our meta-analysis.

### Meta-analysis outcomes

Meta-analyses were conducted according to a prospectively defined analysis plan. Analyses were performed for change between baseline and individual end of treatment of the 14-item HAMA as an observer rating scale of anxiety using the total score, HAMA sub-scores ‘Psychic anxiety’ and ‘Somatic anxiety’ [42] as well as the single HAMA items ‘Anxious mood’, ‘Tension’, ‘Sleep’, ‘Intellectual’, ‘Depressed mood’, ‘Somatic – muscular’, and ‘Somatic – sensory’. Additional analyses were performed on the following outcomes: the total scores of the Zung Self-Rating Anxiety Scale (SAS; [66]), of the Covi Anxiety Scale (CAS; [13]), and the anxiety score of the Hospital Anxiety and Depression Scale (HADS; [65]). These rating scales were applied in different trials (Table 1) and were used in our meta-analysis as subject self-ratings of anxiety. Clinical Global Impressions scale (CGI; [48]) item ‘Global improvement’ was evaluated as a global, observer-rated assessment of change in mental health. The ‘Mental health’ and ‘Physical health’ summary scores of the Short Form Health Survey (SF-36; [62]) administered in 4 out of the 5 trials were included as patient-reported measures of disease-associated quality of life. Moreover, we assessed treatment response defined prospectively as a ≥ 50% reduction of the HAMA total score compared to baseline or a score ≤ 2 (‘much improved’) for CGI item ‘Global improvement’ as well as full remission defined as a HAMA total score < 7 points at the end of the treatment [43].

Tolerability was assessed by analyzing the proportions of subjects in each treatment group with at least one AE, at least one serious AE (SAE), and subjects prematurely withdrawn due to an AE.

### Statistical methods

Meta-analyses were performed on the individual subject data of the included trials that were obtained from the study sponsor. The applicable analysis data sets included the full analysis set (FAS) of the original protocols for all efficacy-related outcomes and the safety analysis set for analyses of AEs as well as for premature withdrawal. For efficacy outcomes, missing data were imputed by carrying forward the last valid observation in order to maintain comparability with the published results of the trials.

To characterize the study populations, descriptive statistics were computed for age, sex, and premature withdrawal rate. Meta-analyses were based on a two-stage approach [10, 50]: within each trial, continuous outcomes were analyzed using analysis of covariance (ANCOVA) with the difference between baseline and end of treatment for the outcome of interest as the dependent variable, treatment as a factor, and the baseline value of the analyzed outcome as a covariate. For the analysis of CGI item ‘Global improvement’, the baseline score of the item ‘Severity of illness’ was used as a covariate. Scores from items and (sub-)scales were analyzed as continuous outcomes. Marginal (adjusted) mean values and their standard deviations were then used as input for a random-effects meta-analysis on the treatment group mean difference. Inverse variance weighting was used for combining the results of the single trials, and the DerSimonian-Laird method was applied for calculating the variance between the trials. Mean differences (MD) were estimated for the continuous outcomes when the same scale was used in all trials. These effect sizes were presented in forest plot visualizations. Additionally, standardized mean differences (SMD) based on bias-corrected Hedges´ g were indicated within the text to provide better comparability with the literature. Where different scales were used (i.e., various anxiety self-rating sores), SMDs were presented in the forest plot.

For binary outcomes, (1) risk ratios (RRs) (response and remission rates) and (2) odds ratios (ORs) (occurrence of AEs, attrition rates), both accompanied by 95% confidence intervals (CIs), were calculated as effect sizes. Meta-analyses of binary efficacy outcomes were performed using random effects models with the inverse variance method. For safety outcomes, fixed effects models were calculated using Mantel–Haenszel weighting for combining the results from single trials.

For the investigation of subgroups, we used mixed effects models assuming random effects within subgroups and a fixed effect across subgroups.

All *p*-values are two-sided; values ≤ 0.05 were considered descriptively significant.

Heterogeneity between the trials was assessed using the *I*^2^ statistic in accordance with the criteria proposed in Deeks et al. [14].

Meta-analyses were computed with R software (version 3.6.0) using functions ‘metacont’ (for continuous outcomes), ‘metabin’ (for binary outcomes), and ‘forest’ included in package meta (version 4.13–0). All other analyses were performed in SAS statistical software version 9.4 for Windows.

## Results

### Study participant characteristics

In the pooled data set including all five trials, a total of 1213 subjects (Silexan 610; placebo 603) were randomized and 1172 (Silexan 587; placebo 585) were analyzed for efficacy in the FAS (Table 2) and 1206 (Silexan 606; placebo 600) for safety. Overall withdrawal rates were low (12% in the Silexan group and 11% in the placebo group).

**Table 2:** Study population baseline characteristics (number and % or mean ± SD)

Trial	Treatment	Randomized	Drop-outs^a^	FAS	Females^b^	Age (years)^b^	HAMA total score^b^
A	Silexan	110	18 (16.4%)	104	73.1%	45.6 ± 11.4	26.8 ± 5.4
	Placebo	111	14 (12.6%)	108	76.9%	46.6 ± 11.3	27.1 ± 5.2
B	Silexan	86	12 (14.0%)	86	72.1%	48.0 ± 11.3	25.5 ± 6.0
	Placebo	84	10 (11.9%)	84	71.4%	46.9 ± 12.7	26.5 ± 6.1
C	Silexan	160	15 (9.4%)	159	66.0%	47.7 ± 12.6	25.7 ± 5.6
	Placebo	158	13 (8.2%)	156	72.4%	47.9 ± 12.6	25.7 ± 5.2
D	Silexan	118	11 (9.3%)	103	76.7%	43.3 ± 11.7	24.6 ± 4.4
	Placebo	113	8 (7.1%)	102	65.7%	45.5 ± 11.5	25.7 ± 5.1
E	Silexan	136	17 (12.5%)	135	70.4%	45.7 ± 11.5	25.8 ± 4.8
	Placebo	137	19 (13.9%)	135	73.3%	44.6 ± 12.3	25.1 ± 4.7
Pooled	Silexan	610	73 (12.0%)	587	71.0%	46.1 ± 11.9	25.7 ± 5.3
	Placebo	603	64 (10.6%)	585	72.1%	46.4 ± 12.2	25.9 ± 5.2

*FAS* full analysis set, *HAMA* Hamilton Anxiety Rating Scale

^a^Based on all randomized patients

^b^Based on full analysis set

In total, 382 patients suffered from subsyndromal anxiety, 315 from MADD and 790 from GAD (FAS). The participants’ age averaged around 46 years. More than ^2^/_3_ of the subjects of all trials were female.

Within each trial, the HAMA total score baseline mean values were essentially comparable between treatment groups, with a mean value difference of 0.2 points for the pooled data set including all trials. Moreover, the average baseline HAMA total scores of subjects with diagnoses characterized by subthreshold anxiety (trials A-C) and of subjects with GAD (trials D, E) were in a comparable range.

### Anxiety: observer rating

**Fig. 1:**
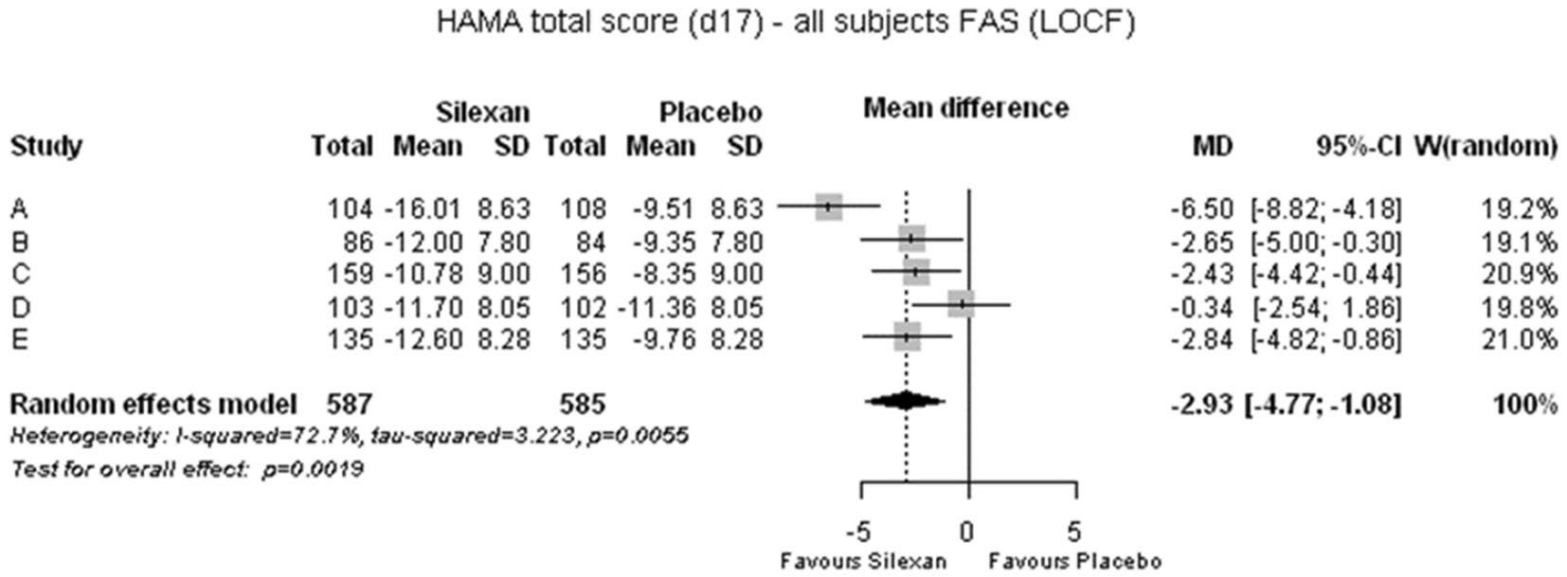
Hamilton Anxiety Scale total score change between baseline and end of treatment (*MD* mean difference, *CI* confidence interval, *W* weight, *FAS* full analysis set, *LOCF* last observation carried forward)

Silexan was significantly more efficacious than placebo in the management of anxiety disorders. Analyzing the HAMA total score reduction, this meta-analysis revealed an average 2.9-point advantage for Silexan over placebo (*p* = 0.002) (Fig. 1), with an estimated minimum benefit of 1.1 points according to the lower bound of the 95% CI. The estimated mean difference corresponds to an SMD of 0.35 (95% CI 0.13; 0.56) favoring Silexan. During the 10-week randomized treatment period, the HAMA total score decreased by averages between 10.8 (trial C) and 16.0 points (trial A) for Silexan and by between 8.4 (trial C) and 11.4 points (trial D) in the placebo groups (Fig. 1).

Figure 1 also indicates that Silexan was significantly superior to placebo in 4 out of the 5 individual trials included in the meta-analysis. Substantial heterogeneity (*I*^2^ = 72.7%) was mainly attributable to between-trial differences in effect sizes favoring Silexan, not to different directions of the treatment effect.

**Fig. 2:**
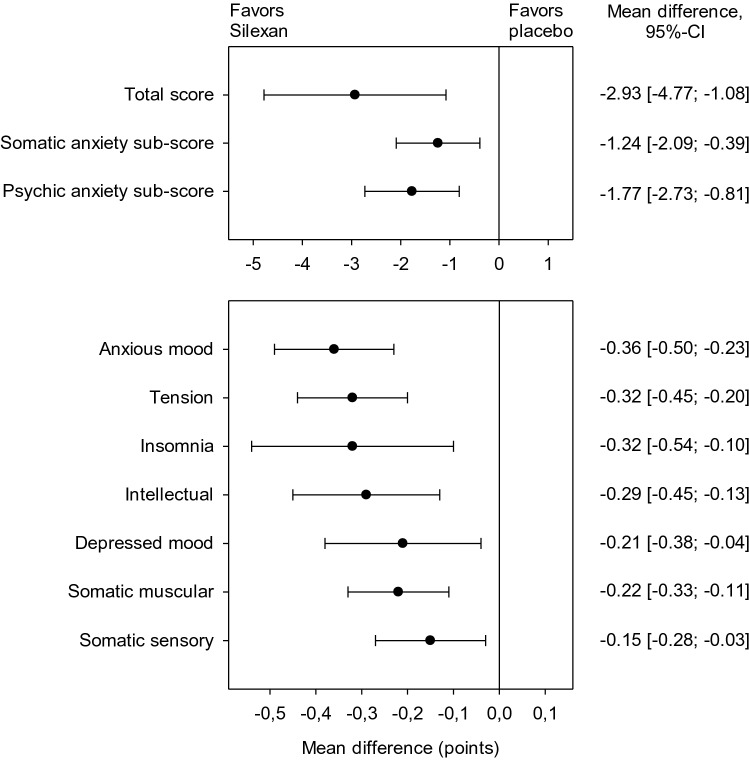
Meta-analysis of trials A–E. Hamilton Anxiety Scale total score, sub-score, and individual item score treatment group differences for change between baseline and end of treatment (random effects model point estimates and 95% confidence intervals)

Figure 2 summarizes the treatment group mean differences and their 95% CIs determined in meta-analyses of the HAMA psychic and somatic anxiety sub-scores (upper panel) and for individual HAMA items identified prospectively in the analysis plan (lower panel). According to the lower bounds of the 95% CIs, Silexan was significantly superior to placebo in reducing anxiety-associated symptoms for both sub-scales as well as for all single HAMA items investigated, with the largest effect sizes observed for anxious mood followed by tension and insomnia.

**Fig. 3:**
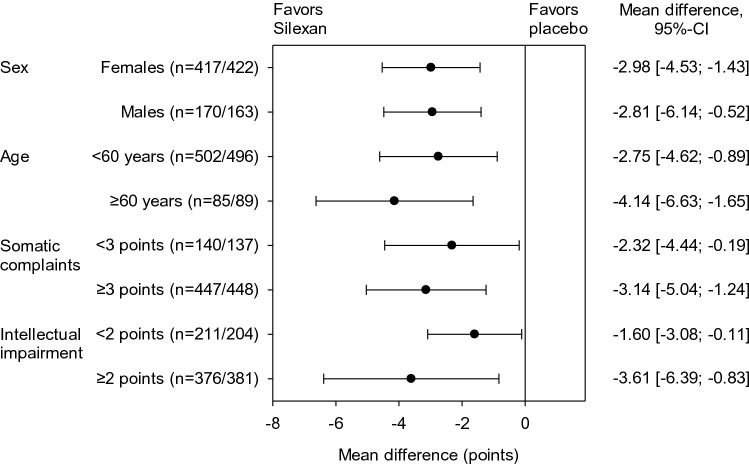
Meta-analysis of trials A–E. Hamilton Anxiety Scale total score treatment group differences for change between baseline and end of treatment in pre-defined subsets of subjects (random effects model point estimates and 95% confidence intervals; number of valid subjects for Silexan/placebo in parentheses)

Main results of subgroup meta-analyses of HAMA total score change broken down by subject sex, age, as well as by somatic complaints at baseline (based on the sum of the scores of HAMA items ‘Somatic muscular’ and ‘Somatic sensory’) and by intellectual impairment (based on HAMA item ‘Intellectual’) are presented in Fig. 3. Whereas the effect sizes for female and male subjects were comparable, elderly subjects showed a larger treatment effect than younger participants, and comparatively larger improvements were also observed for subjects with more pronounced somatic complaints and intellectual impairment (i.e., difficulty in concentration, poor memory) at baseline. Figure 3 also shows that Silexan was significantly superior to placebo in all subsets investigated.

**Fig. 4:**
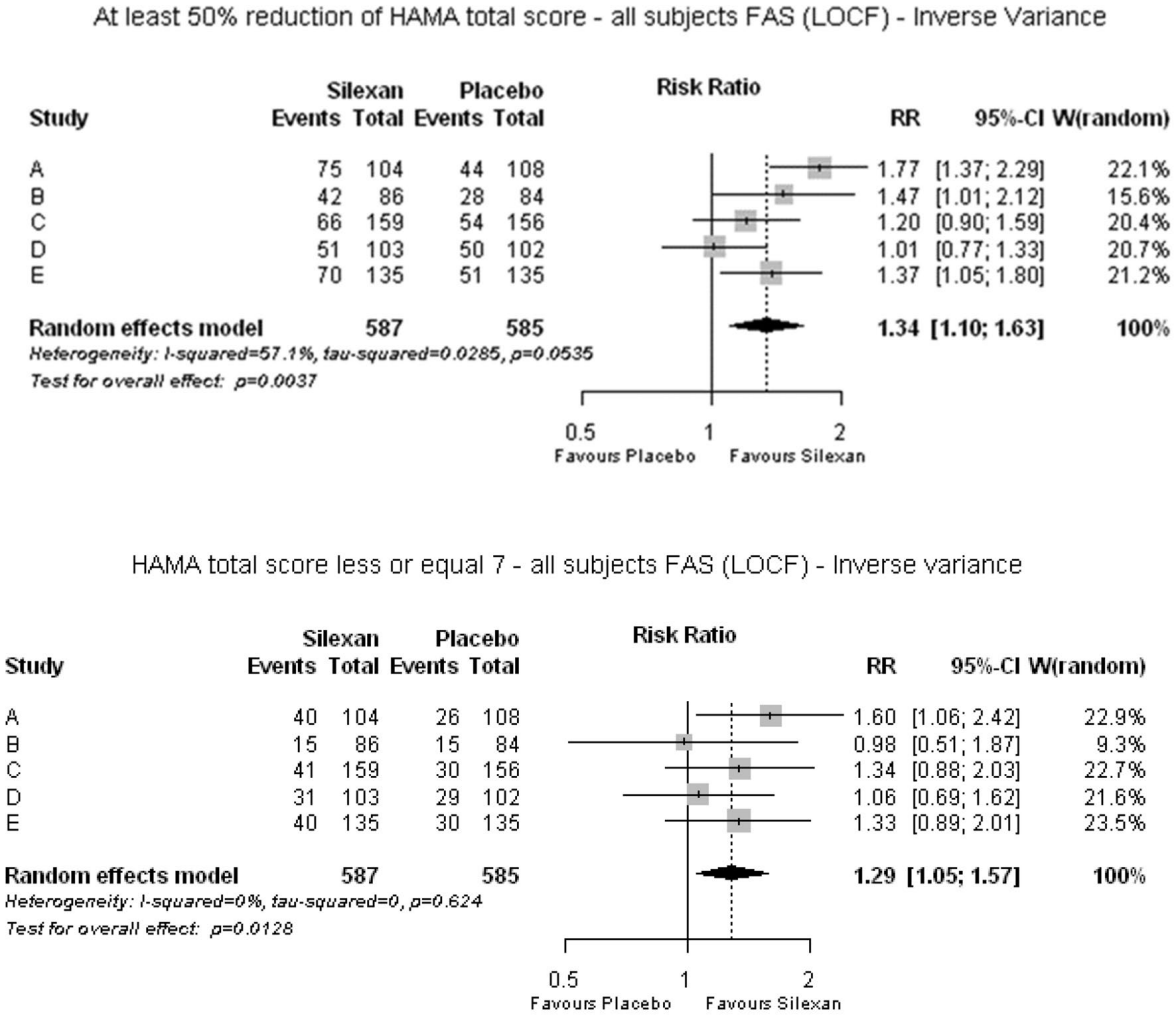
Treatment response (upper panel) and complete remission (lower panel) at end of treatment according to criteria derived from the Hamilton Anxiety Scale total score (*RR* risk ratio, *CI* confidence interval, *W* weight, *FAS* full analysis set, *LOCF* last observation carried forward)

At end of treatment, 304/587 subjects (51.8%) of the Silexan 80 mg/day groups of the 5 trials and 227/585 subjects (38.8%) of the placebo groups showed a HAMA total score reduction by ≥ 50% of their baseline value and were thus classified as treatment responders, with a meta-analysis risk ratio of 1.34 favoring Silexan (*p* = 0.004; Fig. 4). The corresponding number needed to treat (NNT, with 95% CI) was 8 (5; 28) for trials A–E and 6 (4; 36) for trials A-C (performed in subthreshold anxiety). Complete remission (i. e., a HAMA total score < 7 points at end of treatment) was achieved by 167/587 (28.4%) and 130/585 subjects (22.2%) for Silexan and placebo, respectively (meta-analysis risk ratio: 1.29; p = 0.013).

### Anxiety: self-rating

**Fig. 5:**
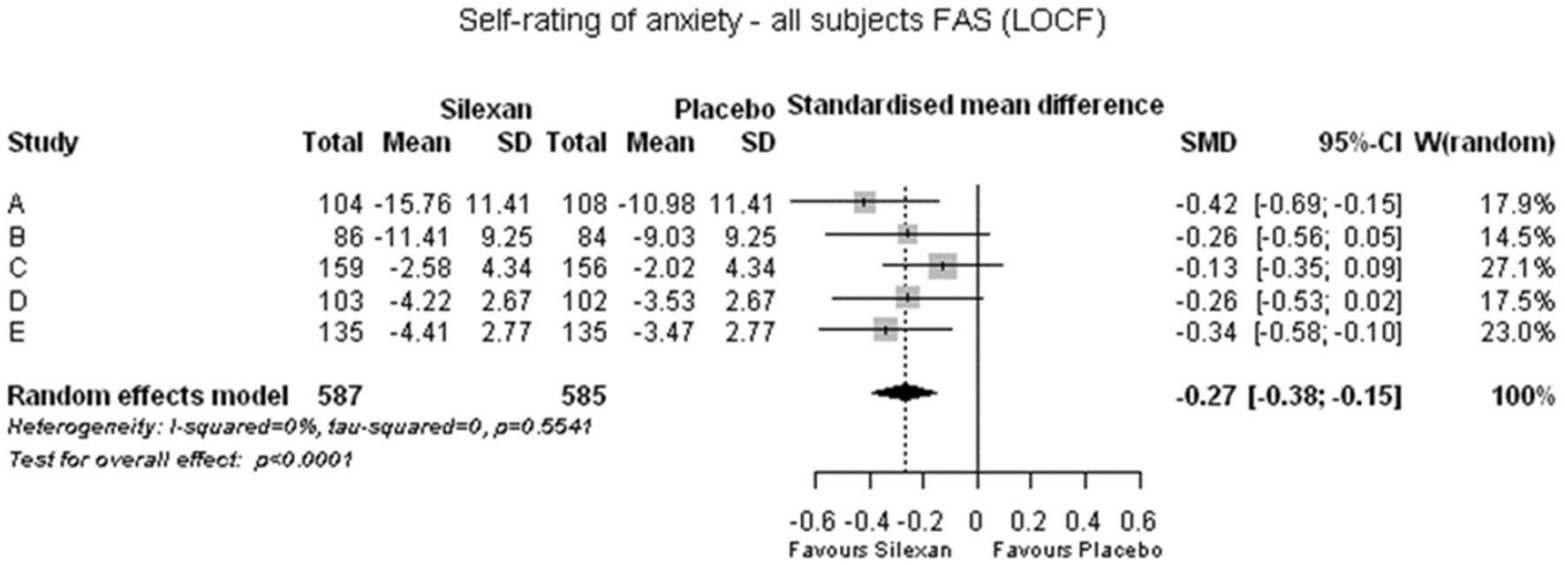
Anxiety self-rating total score change between baseline and end of treatment (*SMD* standardized mean difference, *CI* confidence interval, *W* weight, *FAS* full analysis set, *LOCF* last observation carried forward)

The results of the meta-analysis performed on change from baseline of the total scores of the anxiety self-rating scales (trials A, B: SAS, trial C: HADS, trials D, E: CAS) are presented in Fig. 5. To account for the different ranges of the scales, standardized mean differences (SMDs) based on Hedges’ g with bias correction were calculated as effect size. Significant superiority of Silexan group over placebo was determined with a SMD of 0.27 standard deviation units (*p* < 0.0001). For trial D, showing the lowest effect size (of the included/individual trials) in the observer-rated HAMA (Fig. 1), the standardized mean difference to placebo in the subject self-rating (Fig. 5) differed only marginally from the meta-analysis average across all trials.

### Clinical global impression

**Fig. 6:**
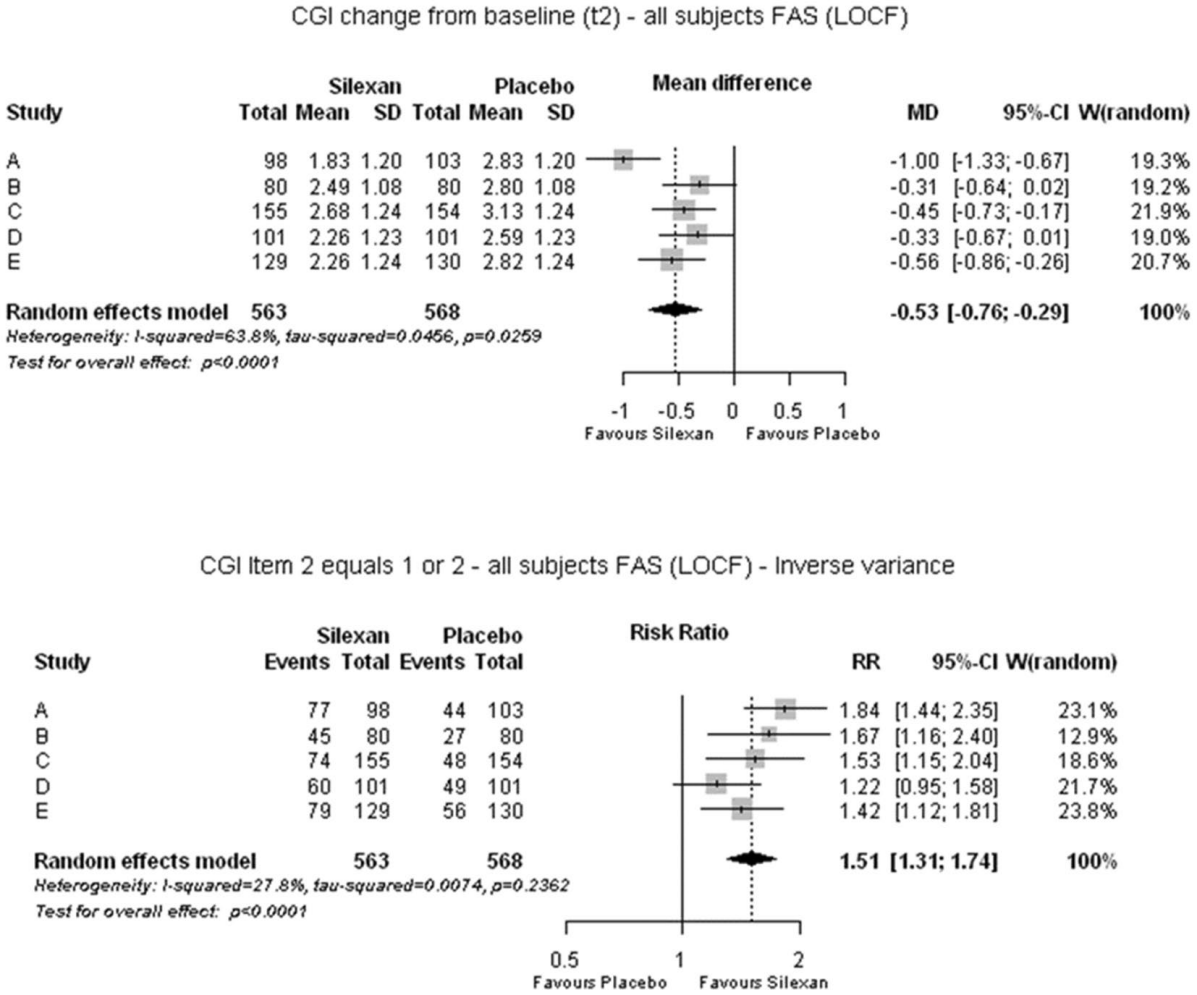
Clinical global impression. Global improvement (upper panel) and responder rate (proportion of subjects much or very much improved; lower panel) at end of treatment (*MD* mean difference, *RR* risk ratio, *CI* confidence interval, *W* weight, *FAS* full analysis set, *LOCF* last observation carried forward)

Analyzing the CGI item ‘Global improvement’ as parameter for the participants’ general mental condition, Silexan 80 mg/day was significantly superior to placebo. (Fig. 6, upper panel; MD = 0.53; *p* < 0.001). The corresponding SMD amounted to 0.44 (95% CI 0.25; 0.63) favoring Silexan. In the accompanying meta-analysis of dichotomous/binary treatment response based on the CGI (Fig. 6, lower panel), Silexan-treated subjects were on average 51% more likely to be much or very much improved than placebo-treated subjects according to the risk ratio (*p* < 0.001), with a pooled responder rates of 59.5% (335/563 subjects) and 39.8% (226/568 subjects) for Silexan and placebo, respectively, and an NNT of 5 (95% CI: 4; 8).

### Disease-associated quality of life

**Fig. 7:**
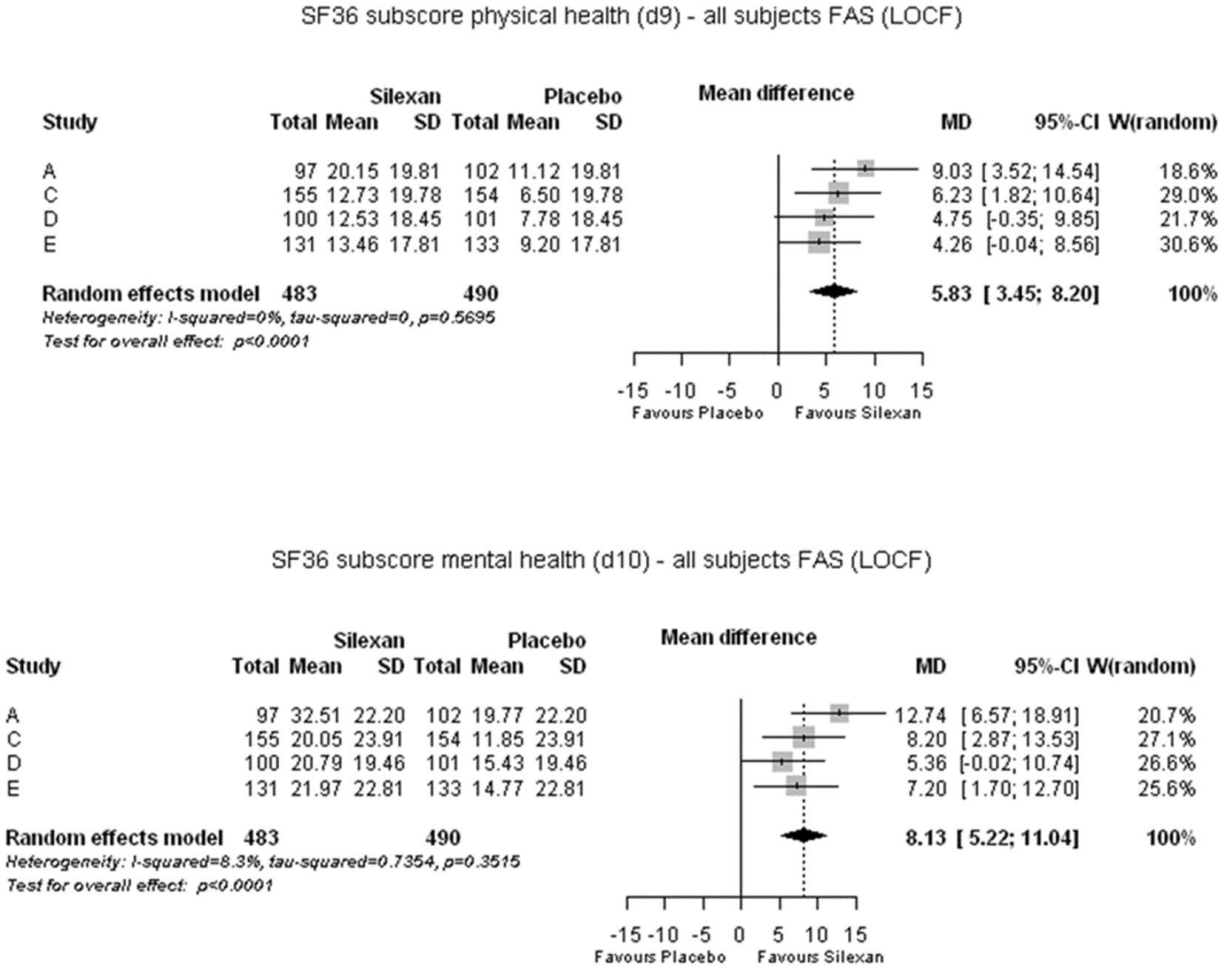
SF-36. Change in summary scores ‘Physical health’ (upper panel) and ‘Mental health’ (lower panel) between baseline and end of treatment (*MD* mean difference, *CI* confidence interval, *W* weight, *FAS* full analysis set, *LOCF* last observation carried forward)

Figure 7 presents the meta-analysis results for the physical (upper panel) and mental health (lower panel) summary scores of the SF-36. Improvements in both domains of patient-reported, disease-associated quality of life were significantly more pronounced for Silexan than for placebo (MD = 5.83 [physical health]; MD = 8.13 [mental health]; *p* < 0.001 for both summary scores), corresponding to a SMD of 0.58 for physical health and of 0.81 for mental health.

### Tolerability

No significant between-group differences for the occurrence of at least one AE (OR = 1.16, 95%-CI 0.91; 1.49, *p* = 0.24, fixed effect model) and at least one SAE (OR = 1.18, 95% CI 0.38; 3.69, *p* = 0.73, fixed effects model) could be determined. SAEs were reported in a total of 6/606 subjects (1.0%) for Silexan and in 5/600 (0.8%) for placebo.

There was no evidence for a difference between groups in the risk of premature withdrawal due to AE (OR = 1.14, 95% CI 0.55; 2.33, *p* = 0.73, fixed effect model). The corresponding attrition rates were 2.6% (16/606 subjects) for Silexan and 2.3% (14/600 subjects) for placebo.

## Discussion

With 14 clinical trials of different development phases published by the end of the year 2020 and a total of about 2,200 subjects evaluated, Silexan is probably the best researched herbal medicinal product for the treatment of subthreshold anxiety, anxiety disorders and related clinical manifestations worldwide [8, 44, 58, 61]. The body of evidence includes 5 double-blind, randomized, placebo-controlled trials in subthreshold anxiety and anxiety disorders, all of which were included into our meta-analysis. Silexan, administered at the marketed dosage of 1 × 80 mg/day, was shown to be significantly superior to placebo in the observer-rated HAMA, including its psychic and somatic anxiety sub-scores and a set of prospectively selected single HAMA items, as well as in different patient-reported anxiety scales. Hence, the anxiolytic effect of Silexan was not exclusively observed by the attending physicians but also subjectively perceived by the study participants. Beyond the indication-specific symptoms assessed by means of the anxiety scales, the herbal medicinal product was also associated with significant improvements in patient's global functioning as assessed by the CGI, indicating a beneficial effect on mental health. Finally, our meta-analysis also showed a significant positive effect of Silexan on patient-reported, disease-associated quality of life, with comparable improvements over placebo in the areas of mental and physical health. The latter results correspond with existing evidence reporting beneficial effects of Silexan on co-occurring depressive symptoms [8], sleep disturbances [58], as well as psychosomatic symptoms as fatigue and pain for instance [61].

Importantly, superiority over placebo was observed for all diagnostic categories represented in our meta-analysis (subthreshold anxiety disorder, MADD, GAD). While our analyses were limited to the marketed Silexan dosage of 1 × 80 mg/day, a pooled analysis of the placebo-controlled trials performed in GAD (trials D and E) published by Kasper et al. [37] showed a clear dose–response relationship for once-daily doses of Silexan between 10 and 160 mg, with 80 mg/day at the lower end of the therapeutic range and a substantially more pronounced anxiolytic effect at 160 mg/day, the maximum dosage investigated in these trials. It is also worth mentioning that 160 mg/day were not associated with a higher rate of AEs than lower dosages of Silexan or placebo [36, 37].

For HAMA total score reduction from baseline, our meta-analysis also indicates significant superiority of Silexan over placebo for prospectively defined subsets of subjects. While women are more frequently affected by anxiety disorders than men [21] and at least ^2^/_3_ of the subjects in each trial were female, our subgroup analysis indicates that subjects of both sexes show a similarly pronounced response to Silexan. For subsets defined by age, it is certainly of clinical interest that treatment with the herbal medicinal product was at least as efficacious in elderly subjects as in younger ones, even though the number of participants aged 60 or above was quite low since patients aged over 65 were excluded from the trials. Similar observations were reported in a meta-analysis by von Känel et al. [61] who found that the effect of Silexan on somatic symptoms and physical health of patients with anxiety disorders was largely independent of age and sex. Consistent with our findings, this meta-analysis could determine a superiority of Silexan over placebo in treating somatic manifestations of anxiety [61].

At baseline, close to ^2^/_3_ of the subjects in the pooled data set suffered from at least moderate, anxiety-related difficulties in concentration and poor memory (labelled ‘Intellectual impairment’ in the HAMA). Our subgroup meta-analysis indicates that subjects initially affected by these difficulties showed improvement of anxiety-associated symptoms, including impaired memory and concentration, and benefited from Silexan treatment to at least the same extent as those with lower or no baseline impairment. This is consistent with other research showing that, unlike several other anxiolytic drugs, Silexan is devoid of central depressant effects [57, 58].

For HAMA total score change, which served as a primary outcome measure of anxiolytic efficacy in all trials available for analysis, no minimal clinically important difference (MCID) has yet been derived empirically, and thus the interpretation of differences between medication and placebo with regard to clinical meaningfulness is not straightforward. According to the protocols of the included trials, a treatment group mean value difference in HAMA total score reduction of between 2.5 and 3.5 points was assumed to be clinically important. The observed meta-analysis mean value difference of 2.93 points was found to be within this range, and 4 out of the 5 analyzed trials showed mean differences between 2.43 and 8.5 points favoring Silexan. Standardized effect size comparisons between anxiolytic agents and placebo for HAMA total score change versus baseline in patients with GAD have been determined in several meta-analyses: Hidalgo et al. [29] (21 trials) reported effect sizes of 0.50 for pregabalin, 0.45 for hydroxyzine, 0.42 for venlafaxine, 0.38 for benzodiazepines, 0.35 for SSRIs, and 0.17 for buspirone. Gomez et al. [23] (56 trials) found effect sizes of 0.33 for SSRIs, 0.34 for SNRIs, 0.50 for benzodiazepines, and of 0.37 for all drug classes combined. In the analysis of Carl et al. [11], meta-analysis effect sizes were 0.38 for psychopharmacotherapy (43 trials) and 0.76 for psychotherapy (39 trials). With an SMD of 0.35, the standardized effect size for Silexan determined in this meta-analysis was thus in the range of the effect sizes for SNRIs and for SSRIs, which are currently recommended as first-line treatment for anxiety disorders [1, 6, 7, 29, 40, 47, 59]. Moreover, when comparing the efficacy of Silexan with that of other medicinal herbs, Silexan yielded the highest effect size when analyzing the herbs with an at least moderate cumulative sample size [64].

There is evidence that reluctance to accept the unwanted effects of conventional anxiolytic agents such as SSRIs, SNRIs and benzodiazepines, together with concerns regarding the sustainability of their anxiolytic effects [52], may contribute to the not seldom occurring under- or even un-treatment of anxiety disorders [12]. AEs attributed to Silexan were mainly limited to mild, transient gastrointestinal effects such as nausea or eructation [63]. The herbal product does, however, not interfere with activities of daily living [34], including driving performance [45], and has neither a sedating effect nor a potential for abuse [57, 58]. Silexan does also not cause withdrawal symptoms even when discontinued abruptly [19] and does not interact with oral contraceptives [28], which is an important finding since an appreciable proportion of patients affected by anxiety disorders and subthreshold anxiety are young female patients [60]. Generally, therapeutic doses of Silexan did not show any interaction potential with activities of cytochrome P450 enzymes CYP1A2, 2C9, 2C19, 2D6, and 3A4 [16].

In anxiety disorders, an appreciable proportion of patients has been shown to respond favorably to placebo treatment [25]. This is also a known issue in placebo-controlled trials in the indication, and placebo response rates have been shown to even rise during the last decades [51]. The results of the individual studies and our meta-analyses in favor of Silexan are remarkably consistent.

As a limitation of this work, all trials available for analysis were initiated by the manufacturer of Silexan and performed in one country (Germany). However, a number of reviews and meta-analyses of the effects of lavender preparations in general and of Silexan in particular has been published independently [15, 30, 54, 63], which underlines the scientific and clinical interest in the application of lavender oil preparations in and beyond anxiety disorders and the robustness and soundness of the results. Although there appears to be a general agreement among the authors that Silexan has a significant and clinically meaningful anxiolytic effect, further independent trials which would investigate the broad potential of its efficacy would be welcome. As a further potential limitation, it should be considered that the diagnoses of the enrolled patients differed between the individual trials. In one trial, participants suffering from mixed anxiety and depressive disorder were recruited [39]. However, in all included trials, patients had to fulfill an anxiety specific inclusion criterion (HAMA total score of ≥ 18 points).

In conclusion, our meta-analysis shows a clinically important anxiolytic effect of Silexan at the marketed dosage of 1 × 80 mg/day in subthreshold anxiety and anxiety disorders, with statistically significant superiority over placebo. A beneficial effect was consistently observed for observer-rated and for patient-reported anxiety, for overall mental condition, as well as for self-rated, disease-associated quality of life. Moreover, significant superiority of Silexan was also stable within several pre-specified subsets of subjects defined by demographic features and baseline characteristics. The analyses do not indicate a systematic, Silexan-associated risk of AEs above the placebo level.

## Data Availability

Raw data cannot be shared both due to ethical reasons and to data protection laws. To the extent permitted by law, the trial data required for validation purposes have already been disclosed in result reports on corresponding databases. All relevant data are within the paper.

## Footnotes

1 Silexan® is the proprietary active ingredient of Dr. Willmar Schwabe GmbH & Co. KG, Karlsruhe, Germany.
